# Operational Ethics in the Cardiovascular Intensive Care Unit: A Practical Framework for Ethical Decision-Making

**DOI:** 10.3390/jcm15176885

**Published:** 2026-09-05

**Authors:** Lourdes Vicent, Rafael Salguero-Bodes, Pablo R. Alonso, Elena Puerto, Carlos Diaz-Arocutipa, Fernando Arribas Ynsaurriaga, Roberto Martín-Asenjo

**Affiliations:** 1 Department of Cardiology, Hospital Universitario 12 de Octubre, 28041 Madrid, Spain; rafael.salguero@salud.madrid.org (R.S.-B.); prodriguezalonso@hotmail.com (P.R.A.); elenapuerto.garcia@salud.madrid.org (E.P.); fernando.arribas@salud.madrid.org (F.A.Y.); rmartina@salud.madrid.org (R.M.-A.); 2 Instituto de Investigación Sanitaria Hospital 12 de Octubre (imas12), 28041 Madrid, Spain; 3 Centro de Investigación Biomédica en Red Enfermedades Cardiovasculares (CIBERCV), 28029 Madrid, Spain; 4 Facultad de Medicina, Universidad Complutense, 28040 Madrid, Spain; 5 Comité de Ética de la Investigación con Medicamentos, Hospital 12 de Octubre, 28041 Madrid, Spain; 6 Unidad de Revisiones Sistemáticas y Meta-Análisis (URSIGET), Vicerrectorado de Investigación, Universidad San Ignacio de Loyola, Lima 15024, Peru; carlosdiaz013@gmail.com

**Keywords:** cardiovascular intensive care unit, cardiovascular critical care, clinical ethics, ethical decision-making, operational ethics, mechanical circulatory support, extracorporeal membrane oxygenation, end-of-life care, moral distress, shared decision-making

## Abstract

**Background/Objectives:** Contemporary cardiovascular critical care is increasingly shaped by advanced technologies, prognostic uncertainty, and time-sensitive decisions regarding mechanical circulatory support, treatment limitation, device management, family communication, and end-of-life care. Although established ethical principles provide essential normative guidance, their integration into routine bedside workflows remains challenging. This review examines the principal ethical tensions arising in the Cardiovascular Intensive Care Unit (CICU) and proposes a practical framework for real-time ethical decision-making. **Methods:** A narrative review of the contemporary literature was performed using major biomedical databases together with international guidelines, scientific statements, consensus documents, and relevant ethics literature to identify the principal ethical challenges encountered in contemporary cardiovascular intensive care and to develop an implementation-oriented framework for integrating ethical reasoning into routine clinical workflows. **Results:** Ethical challenges in the CICU frequently arise during transitions between therapeutic escalation, reassessment, limitation, withdrawal, and comfort-focused care. These decisions are influenced not only by clinical prognosis but also by organisational culture, multidisciplinary dynamics, patient preferences, communication, and structural inequities. We propose Operational Ethics as an implementation framework that embeds ethical reflection into routine clinical workflows through continuous reassessment of therapeutic goals, proportionality, prognosis, patient values, and multidisciplinary deliberation. Practical strategies include ethical pause points, time-limited trials, structured communication pathways, anticipatory device discussions, regular goals-of-care reassessment, and institutional support for clinicians experiencing moral distress. **Conclusions:** Operational Ethics complements established bioethical principles by translating them into practical bedside processes adapted to the temporal and organisational realities of cardiovascular critical care. Its integration into routine CICU practice may support more consistent, equitable, and patient-centred decisions while ensuring that technological possibilities remain aligned with meaningful clinical goals.

## 1. Introduction

The modern Cardiovascular Intensive Care Unit (CICU) has evolved far beyond its original role as a coronary care ward focused on arrhythmia monitoring and rapid reperfusion [1,2]. It is now a highly technologized environment where decisions about mechanical circulatory support, device activation or deactivation, and therapeutic escalation or limitation frequently occur under substantial time pressure and prognostic uncertainty [3]. Although the organisation and ethical practices of CICUs vary considerably across institutions and healthcare systems, these environments share common challenges arising from urgency, technological complexity, and the need to make high-stakes decisions under conditions of uncertainty. As technological capacities increase, many interventions acquire greater ethical complexity because they must balance potential benefit, treatment burden, prognostic uncertainty, and patient preferences. The CICU may therefore represent a particularly ethics-intensive clinical environment, although the frequency and nature of these challenges vary across institutions and healthcare systems [4,5].

Ethical dilemmas are therefore not limited to rare or extraordinary situations. Rather, they may emerge repeatedly during routine clinical practice whenever uncertainty, competing goals, and rapidly changing physiology converge. Decisions such as initiating ECMO in patients with uncertain neurological prognosis, replacing an implantable cardioverter–defibrillator (ICD) in those with limited life expectancy, deactivating devices delivering shocks at the end of life, or responding to families demanding “everything possible” during CPR are not merely technical—they shape trajectories of suffering, dignity, and death in real time [6,7].

Access to the CICU itself may represent one of the earliest ethically relevant decisions in a patient’s trajectory [7]. Bed allocation, ECMO team activation, and candidacy for mechanical support are primarily guided by clinical criteria such as severity, reversibility, expected benefit, comorbidities, and resource availability. However, observational and qualitative evidence suggests that implicit or structural factors—including age, sex, frailty, socioeconomic circumstances, mental health, communication barriers, and family advocacy—may also influence these processes [8,9]. Many of these ethical tensions became particularly evident during the COVID-19 pandemic, when resource constraints, delayed STEMI presentation, and healthcare professional burnout exposed the vulnerability of cardiovascular critical care systems [10]. In this review, we use the term “hidden moral triage” to describe the potential influence of such insufficiently explicit or undocumented factors on selection and allocation decisions, rather than to imply that these decisions are routinely discriminatory or clinically unjustified.

Over the last decade, ethical guidance, multidisciplinary shock teams, and the integration of palliative care into cardiovascular critical care have advanced considerably. However, the extent to which these resources are incorporated into routine CICU workflows remains variable across institutions and healthcare systems. Existing ethical frameworks provide essential normative guidance through principles such as autonomy, beneficence, non-maleficence, and justice, but they may offer less specific direction regarding how ethical reasoning should be operationalised during rapidly evolving bedside decisions [9,11]. Thus, the challenge is not an absence of ethical infrastructure, but the need to adapt and integrate existing ethical resources alongside increasingly complex cardiovascular technologies and time-sensitive clinical pathways.

This narrative review examines recurrent ethical challenges arising from therapeutic escalation, treatment limitation, allocation of advanced therapies, device management, communication, research, and professional moral distress in the contemporary CICU. It proposes Operational Ethics as an implementation-oriented framework for integrating established ethical principles into time-sensitive clinical workflows. Although examples throughout the review are primarily drawn from European and North American cardiovascular critical care literature, the proposed framework is intended to be adaptable across different healthcare systems. Its practical implementation should be interpreted within the legal, cultural, organisational, and resource contexts of individual institutions and jurisdictions. The conceptual scope of the framework is defined in the following subsection.

### 1.1. Conceptual Definition and Scope of Operational Ethics

Operational Ethics is proposed as an implementation-oriented framework that translates established ethical principles into routine bedside decision-making within the Cardiovascular Intensive Care Unit (CICU). It is not intended as a new ethical theory or as a replacement for principlism, clinical ethics consultation, palliative care, or shared decision-making. Rather, it provides a practical structure for integrating ethical reflection into time-sensitive clinical workflows. Operational Ethics should not be understood as an alternative to existing clinical or organisational practices. Palliative care consultation, formal ethics consultation, multidisciplinary rounds, shock-team decision-making, family meetings, and time-limited trials each fulfil distinct and complementary roles within contemporary cardiovascular critical care. Operational Ethics does not replace these processes; rather, it provides an implementation framework that connects them through continuous ethical reflection embedded within routine clinical workflows. In this sense, it focuses less on creating new structures than on ensuring that ethical reasoning accompanies everyday clinical decisions across existing organisational processes.

Unlike traditional bioethical frameworks, which primarily define what constitutes ethically appropriate care, Operational Ethics focuses on how and when ethical reasoning can be incorporated into real-time decisions. It emphasises continuous reassessment of therapeutic goals, proportionality, patient values, multidisciplinary deliberation, and transparent documentation throughout the patient’s clinical trajectory.

Accordingly, Operational Ethics should be understood as an implementation framework that complements existing ethical principles and institutional practices by embedding ethical deliberation into the organisational and temporal realities of contemporary cardiovascular critical care.

### 1.2. Methods

This manuscript was conducted as a narrative review aimed at synthesising the contemporary literature addressing ethical challenges in the Cardiovascular Intensive Care Unit (CICU) and developing a practical framework for integrating ethical reasoning into routine cardiovascular critical care.

A literature search was performed using PubMed/MEDLINE, Scopus, and Web of Science. The literature search was conducted up to July 2026, with primary emphasis on contemporary literature. No strict lower date limit was applied, allowing the inclusion of earlier landmark publications when considered conceptually relevant. Search terms included combinations of “cardiovascular intensive care unit”, “cardiac intensive care”, “clinical ethics”, “bioethics”, “mechanical circulatory support”, “ECMO”, “Impella”, “ventricular assist device”, “device deactivation”, “do-not-resuscitate”, “treatment limitation”, “end-of-life care”, “shared decision-making”, “palliative care”, “moral distress”, “healthcare disparities”, “artificial intelligence”, and “organ donation”.

Additional relevant publications were identified through manual review of the reference lists of key articles. Sources considered for inclusion comprised clinical practice guidelines, scientific statements, expert consensus documents, position papers, observational and other empirical studies, qualitative studies, and relevant clinical ethics analyses. Particular attention was given to documents from major cardiovascular, intensive care, and bioethics societies.

Given the narrative nature of this review, no formal systematic review methodology, risk-of-bias assessment, or quantitative synthesis was performed. Publications were selected according to their scientific relevance, methodological quality, clinical applicability, and contribution to understanding the ethical challenges encountered in contemporary CICU practice. Particular emphasis was placed on evidence capable of informing practical bedside decision-making and on documents with direct implications for cardiovascular critical care.

Source selection was iterative and guided by relevance to the predefined clinical and ethical domains of the review. When several sources addressed the same issue, priority was given to contemporary guidelines and consensus documents, higher-quality empirical evidence, and publications with direct applicability to cardiovascular critical care. The final selection and interpretation of key sources were agreed upon among the authors during manuscript development and revision.

## 2. The CICU as a New Ethical Territory

The evolution of the Cardiovascular Intensive Care Unit (CICU) has transformed not only cardiovascular care but also the ethical context in which clinical decisions are made. The first coronary care units were designed with a singular objective: monitor lethal arrhythmias and intervene rapidly. Their ethical landscape was comparatively straightforward, largely centred on immediate life-saving interventions. Today’s CICU, however, has evolved into a hybrid clinical environment that combines advanced cardiovascular technologies, transplant pathways, prolonged organ support, and increasingly complex end-of-life decision-making [12].

Compared with many general intensive care units, the CICU has traditionally been associated with a strong culture of rescue and technological possibility. The presence of ECMO (Extracorporeal Membrane Oxygenation), ventricular assist devices, invasive haemodynamic monitoring, and rapid reperfusion strategies may naturally favour therapeutic escalation whenever potentially reversible conditions are identified. In this context, continuing or escalating treatment may sometimes appear more intuitive than considering treatment limitation, particularly when advanced technologies remain available [13]. Rather than simply extending life, these technologies may also reshape the ethical context in which decisions are made, making non-escalation feel less like a routine clinical option and more like an active decision requiring explicit justification [14].

The CICU is also a space of selection and exclusion. Access to a specialised cardiovascular intensive care bed, ECMO, or Impella^®^ support is generally presented as a clinical decision based on severity, reversibility, and expected benefit. However, accumulating evidence suggests that patient selection may also be influenced by less explicit factors, including age, frailty, sex, socioeconomic circumstances, communication barriers, mental health, and the availability of family advocacy [15]. These influences are rarely intentional, yet they may shape decisions before formal ethical deliberation or informed consent begins. In this sense, some of the earliest ethical decisions in cardiovascular critical care occur before they are recognised as ethical decisions.

The increasing availability of advanced life-sustaining technologies may also generate a form of therapeutic momentum. A patient receiving escalating vasopressor support may rapidly become a candidate for mechanical circulatory support, and once ECMO is actively considered or initiated, subsequent decisions often shift from whether escalation is appropriate to how long escalation should continue [13]. This phenomenon—described as technological momentum or technological inertia—does not imply inappropriate care; rather, it reflects the psychological, organisational, and emotional challenges of transitioning from life-prolonging treatment to treatment limitation once advanced support has been established [8,16].

This culture of escalation also shapes professional development. Young cardiologists are trained to respond rapidly to life-threatening situations in which delayed intervention may have immediate consequences. Although this mindset is fundamental to high-quality acute cardiovascular care, it may also foster a tendency toward early intervention, making deferred decision-making, task delegation, or watchful waiting psychologically more difficult in selected clinical situations [12].

## 3. Time Pressure, Monitoring, and the Acceleration of Ethical Decisions in the CICU

The CICU is governed by a temporal logic that differs substantially from many other clinical environments. Although important therapeutic decisions are frequently discussed within multidisciplinary teams and increasingly supported by structured shock pathways, many high-acuity situations still require clinicians to act within minutes while simultaneously interpreting rapidly evolving physiological information. Decision-making therefore often occurs under severe time constraints, driven by a continuous stream of haemodynamic data, invasive pressure tracings, oxygen saturation trends, lactate measurements, and vasopressor requirements [17]. These data streams continuously demand clinical interpretation, potentially reducing opportunities for explicit ethical reflection during acute deterioration [17].

When clinical deterioration occurs, teams must frequently balance two parallel processes: rapid physiological stabilisation and ethical deliberation. Escalation strategies—including advanced circulatory support, emergency coronary intervention, or mechanical ventilation—are supported by well-established operational pathways. Ethical processes, however, often require a different rhythm, involving communication with patients and families, multidisciplinary discussion, prognostic reassessment, and consideration of patient values and goals of care. The challenge is therefore not that ethical reflection is absent from contemporary CICU practice, but that it must often occur simultaneously with urgent clinical action rather than preceding it [18].

In many situations, clinical actions may acquire ethical significance before they are explicitly recognised as ethical decisions. Preparing an ECMO circuit, mobilising a shock team, or opening an Impella^®^ device may progressively increase both procedural and psychological commitment toward therapeutic escalation. Once advanced support has been initiated, clinicians and families frequently face a different ethical question: not whether escalation was appropriate, but under which circumstances ongoing support should be continued, reassessed, or withdrawn. This asymmetry reflects the phenomenon described in the literature as technological momentum, whereby initiating highly invasive therapies may influence subsequent decision-making beyond purely physiological considerations [13,19,20].

The increasing integration of prognostic scores, clinical algorithms, and artificial intelligence (AI)-supported tools introduces an additional ethical dimension into CICU decision-making. AI is increasingly being explored for early detection of deterioration, cardiogenic shock prediction, ICU and neurological prognostication, and assessment of candidacy for advanced therapies such as ECMO. Although these tools may improve risk stratification and consistency, their use raises concerns regarding algorithmic bias, limited external validation, explainability, automation bias, and accountability. Models trained on historical or non-representative data may reproduce existing inequities, while apparently objective numerical outputs may receive disproportionate weight under time pressure. AI should therefore support rather than replace clinical judgement. When an algorithmic recommendation conflicts with bedside assessment, responsibility remains with the treating team, which must consider the model’s applicability, uncertainty, patient values, and the ethical consequences of the final decision [19,21].

Structured pathways and multidisciplinary review may improve consistency, but the final decision must remain contextual, clinically accountable, and responsive to patient values (Table 1) [22].

## 4. Initiation, Continuation and Withdrawal of Advanced Cardiovascular Support

### 4.1. ECMO, Impella^®^, IABP: Rescue Therapy or Prolongation of Dying

Few interventions embody the ethical complexity of contemporary cardiovascular critical care as clearly as advanced mechanical circulatory support. ECMO, Impella^®^, and intra-aortic balloon pump (IABP) are primarily intended as temporary therapies that provide time for myocardial recovery, definitive treatment, transplantation, durable mechanical support, or informed clinical decision-making. For many patients, these technologies successfully fulfil these objectives. However, in a subset of patients who fail to recover or become ineligible for further therapeutic options, the original purpose of support may progressively become less clear.

This transition raises ethical questions that extend beyond technical management. When native cardiac function is replaced by mechanical circulatory support, physiological stability may persist despite irreversible neurological injury, progressive multiorgan dysfunction, or the absence of a realistic recovery pathway. In these circumstances, clinicians and families may experience uncertainty regarding whether ongoing support continues to serve a therapeutic purpose or primarily prolongs physiological function. These principles are consistent with recent ELSO ethical guidance [23,24], which emphasises careful patient selection, explicit therapeutic goals, bridge-to-decision strategies, multidisciplinary shock-team evaluation, and predefined criteria for reassessment or withdrawal when treatment no longer provides proportionate benefit.

This situation has been described as technological inertia or technological momentum, whereby the initiation of highly invasive support may make subsequent treatment limitation psychologically, emotionally, and organizationally more difficult, even when clinical goals become unattainable [25].

Accordingly, the ethical question is not whether advanced support should be initiated, but how clinicians can continuously reassess whether the goals that originally justified treatment remain achievable as the patient’s clinical trajectory evolves. In selected patients, advanced mechanical circulatory support may therefore accompany not only recovery but also the process of dying, creating uncertainty about when continued technological support no longer provides proportional clinical Benefit [26] (Table 2).

### 4.2. Time-Limited Trials as an Ethical Tool

Time-limited trials (TLTs) have increasingly been proposed as a practical strategy to address the ethical uncertainty surrounding advanced mechanical circulatory support. Rather than committing to open-ended treatment, TLTs establish predefined clinical objectives, timeframes, and criteria for reassessment before support is initiated. In doing so, they translate the ethical principle of proportionality into a structured clinical process [27,28]. However, their successful implementation depends not only on predefined stopping criteria but also on an institutional culture that supports regular reassessment and accepts treatment limitation as an integral component of high-quality critical care rather than as therapeutic failure.

For TLTs to be meaningful, they require early communication with patients and families whenever possible, explicit documentation of treatment goals, and a shared understanding that discontinuing support after predefined objectives are no longer achievable represents a clinically and ethically appropriate decision rather than abandonment [28].

### 4.3. The Ethical Asymmetry Between Starting and Withdrawing Support

Although initiation and withdrawal of advanced mechanical circulatory support are ethically equivalent when guided by proportionality and patient-centred goals, they are often experienced differently by clinicians, patients, and families. Initiating ECMO or Impella^®^ is commonly associated with the possibility of recovery and therapeutic hope, whereas treatment withdrawal may be perceived as emotionally more difficult because it occurs after a visible therapeutic commitment has already been made [29,30].

Importantly, contemporary CICUs increasingly rely on multidisciplinary shock teams, repeated prognostic reassessment, and shared decision-making throughout the course of treatment. Nevertheless, once advanced support has been established, decisions regarding continuation or withdrawal frequently involve additional emotional, psychological, and organisational challenges that extend beyond the initial decision to initiate therapy. The practical implementation of these principles may vary according to national legislation, institutional policies, cultural values, and the preferences of patients and surrogate decision-makers. Operational Ethics therefore provides a flexible implementation framework rather than a prescriptive model for withdrawal decisions.

Language itself may reinforce this asymmetry. Expressions such as “turning off” or “disconnecting” naturally focus attention on the moment of withdrawal, potentially obscuring the ethical significance of the earlier decision to initiate highly invasive treatment without clearly defined therapeutic goals. From an ethical perspective, both decisions are part of the same continuum of care and should be guided by identical principles of proportionality, patient preferences, expected benefit, and ongoing reassessment.

### 4.4. From Life-Support to Organ Donation: Ethical Transition and the Body as Resource

The increasing implementation of controlled donation after circulatory death (DCD) has introduced new ethical dimensions into contemporary cardiovascular critical care. In selected patients, the CICU becomes the setting in which the transition from life-sustaining treatment to organ donation is considered after recovery is no longer regarded as achievable [31,32].

This transition is ethically significant because the clinical purpose of advanced technological support may progressively change. Devices initially deployed to preserve the possibility of recovery may subsequently contribute to preserving organ viability once treatment goals have shifted toward end-of-life care and donation. Although this transition is supported by well-established ethical and legal frameworks, it may nevertheless generate emotional and moral complexity for clinicians, patients’ families, and healthcare teams.

For families, this transition requires understanding that decisions regarding treatment limitation and decisions regarding organ donation remain ethically distinct, even when they occur within the same clinical trajectory. For clinicians, it may involve navigating a change in professional focus—from pursuing recovery whenever possible to ensuring that end-of-life care, patient dignity, and, where appropriate, organ donation are conducted with equal respect and transparency.

Ethically robust practice requires a clear separation between the decision to withdraw life-sustaining treatment and the subsequent consideration of donation, together with voluntary communication, respect for previously expressed patient preferences, and acknowledgement of the emotional burden experienced by families and professionals [33]. Operational Ethics may support this transition by clarifying the change in therapeutic goals, the responsibilities of the different teams, and the rationale documented at each stage [33].

Additional ethical challenges surrounding controlled donation after circulatory death continue to evolve. Ongoing debates include the use of normothermic regional perfusion, variability in national legislation governing DCD, differing criteria for the determination of death, preservation of public trust in the donation process, and the need to maintain clear professional boundaries between the treating team and transplant teams. Although these issues extend beyond the scope of this review, acknowledging them reinforces that ethically robust donation requires not only legal compliance but also procedural transparency, role separation, and sustained public confidence. Operational Ethics may provide a practical framework for supporting these safeguards during complex end-of-life transitions.

## 5. Goals of Care, Do-Not-Resuscitate Orders, and Therapeutic Limitation in the Contemporary CICU

The contemporary CICU has been built around rapid recognition of deterioration and timely therapeutic intervention. This rescue-oriented culture has transformed outcomes for critically ill cardiovascular patients and remains one of the defining strengths of modern cardiac critical care. At the same time, the increasing availability of advanced therapies has introduced new ethical challenges regarding when treatment should be escalated, continued, or appropriately limited.

Within this environment, decisions regarding Do-Not-Resuscitate (DNR) orders, non-escalation of treatment, and end-of-life care are no longer exceptional events but integral components of comprehensive cardiovascular critical care. The ethical challenge lies not in choosing between treatment and no treatment, but in ensuring that therapeutic intensity remains aligned with patient values, expected benefit, and proportionality throughout the patient’s clinical trajectory.

### 5.1. Integrating Goals of Care into a Rescue-Oriented Environment

The organisational tempo of the CICU prioritises rapid assessment and intervention. Although multidisciplinary shock teams, palliative care integration, and advance care planning have become increasingly incorporated into cardiovascular critical care, opportunities for anticipatory discussions regarding goals of care may remain limited when patients deteriorate rapidly or prognostic uncertainty is high [34].

Consequently, DNR and non-escalation decisions may still occur relatively late during the clinical course of selected patients, sometimes under considerable emotional pressure and within evolving clinical circumstances.

Similarly, documentation of DNR orders should be understood as more than an administrative requirement. When clearly documented and openly discussed, these decisions become communication tools that facilitate shared understanding among clinicians, patients, and families, helping to ensure that future interventions remain consistent with previously established therapeutic goals [35].

### 5.2. Language and the Ethics of Escalation

Language shapes how clinical decisions are understood by healthcare professionals, patients, and families. Expressions such as initiate, escalate, or upgrade naturally convey therapeutic possibility, whereas terms including withdraw, limit, or de-escalate often carry greater emotional weight.

This linguistic asymmetry does not determine ethical decisions, but it may influence how clinicians experience them. Escalation is frequently perceived as pursuing opportunity, whereas treatment limitation may be experienced as accepting clinical limits despite identical ethical foundations. Recognising this difference in language may help explain why decisions that are ethically equivalent are often experienced differently in clinical practice [36] (Table 3).

### 5.3. The Ethical Value of Non-Escalation

Choosing not to escalate therapy may require as much clinical judgement and ethical deliberation as initiating advanced support. Decisions not to proceed from vasopressor therapy to ECMO, not to replace an implantable cardioverter–defibrillator, or not to perform reintubation are active therapeutic decisions based on proportionality, expected benefit, and patient-centred goals.

Although these decisions are increasingly supported by multidisciplinary teams, ethics consultation, and palliative care services, clinicians may nevertheless experience considerable emotional responsibility, particularly when uncertainty persists or disagreement exists among healthcare professionals or family members.

Non-escalation should therefore be documented as an active therapeutic decision rather than as an absence of care [36].

### 5.4. End-of-Life Care Within a High-Technology Environment

End-of-life care in the CICU frequently occurs within an environment characterised by continuous physiological monitoring, advanced technologies, and rapid clinical intervention. This organisational context may make the transition from curative treatment to comfort-focused care particularly challenging [37].

High-quality end-of-life care requires an explicit transition from disease-directed intervention to symptom relief, family support, and dignified care, with the rationale and treatment limits clearly documented.

The presence of a DNR order should not signal the end of clinical care, but rather the beginning of a different form of care—one that prioritises comfort, presence, communication, and respect for patient values over further technological intervention [38,39]. In this sense, the contemporary CICU must combine technological excellence with the capacity to provide compassionate and ethically coherent end-of-life care.

### 5.5. Operationalising Contemporary Ethical Guidance: The ERC 2025 Perspective

The 2025 European Resuscitation Council (ERC) Guidelines on Ethics and End-of-Life Decisions represent a major advance in integrating ethical principles into acute cardiovascular care. Their recommendations emphasise advance care planning, shared decision-making, transparent communication, family involvement, and early recognition of medical futility as integral components of high-quality resuscitation practice [40].

The ERC recommendations provide the normative basis for anticipatory planning, shared decision-making, and treatment limitation. Their application to time-sensitive CICU decisions can be supported by the structured bedside process presented in the Operational Ethics Toolkit [41,42].

The ERC guidelines also emphasise justice and equity, recognising that age, sex, frailty, and social circumstances may influence discussions regarding resuscitation and treatment limitation. Embedding these principles into routine CICU practice therefore represents not only an ethical refinement but also an opportunity to reduce structural inequities in access to advanced cardiovascular care [40].

## 6. Device Ethics: Deactivation and Replacement of ICDs and Ventricular Assist Devices (VAD)

Implantable cardioverter–defibrillators (ICDs), ventricular assist devices (VADs), and other implantable cardiovascular technologies have transformed the prognosis of patients with advanced cardiovascular disease. These devices provide continuous monitoring and life-sustaining therapies that may remain active for many years, often across changing stages of illness and evolving patient priorities. As a result, the ethical challenge is not limited to deciding when these technologies should be implanted, but also when their continued function remains aligned with the patient’s goals, prognosis, and preferences.

### 6.1. The Paradox of the “Automatic Lifesaver”

ICDs are designed to prevent sudden cardiac death and have substantially improved survival in appropriately selected patients. However, during advanced or terminal illness, continued ICD therapies may become inconsistent with comfort-focused goals of care, potentially exposing patients to painful shocks that no longer provide meaningful clinical benefit [43,44,45].

Although current professional societies recommend discussing ICD deactivation as part of advance care planning, these conversations remain challenging in clinical practice. Patients, families, and healthcare professionals may experience deactivation as emotionally difficult because the device has become closely associated with survival and protection. This psychological association may contribute to delayed discussions despite broad ethical and legal consensus that deactivation represents withdrawal of a medical treatment rather than an act intended to hasten death [46].

Operational ethics therefore encourages anticipatory conversations regarding future device management, allowing decisions about deactivation to be incorporated into longitudinal goals-of-care discussions rather than deferred until the final stages of illness.

### 6.2. Replacement Decisions: Between Maintenance and Futility

Generator replacement for ICDs or pacemakers is frequently perceived as a routine procedural step. Nevertheless, every replacement represents a new clinical decision requiring reassessment of life expectancy, functional status, competing comorbidities, expected benefit, and patient preferences [47].

Automatic replacement based solely on battery depletion may overlook substantial changes in prognosis that have occurred since the original implantation. Rather than viewing generator replacement as continuation of previous treatment, clinicians should approach it as an opportunity to re-evaluate whether the goals that originally justified device therapy remain appropriate.

Operational ethics therefore supports structured conversations before generator replacement, integrating clinical prognosis with patient values and documenting the intended goals of continued device therapy. This approach is consistent with recent HRS and EHRA consensus documents, which recommend proactive shared decision-making before generator replacement or elective device interventions [45].

### 6.3. Deactivation as an Active Clinical Decision

Professional discomfort surrounding device deactivation frequently reflects the emotional difficulty of withdrawing an established therapy rather than uncertainty regarding its ethical legitimacy. Current ethical and legal frameworks consistently recognise discontinuation of a non-beneficial cardiovascular device as an appropriate expression of patient autonomy and proportional care [46].

Despite this consensus, clinicians may still experience considerable emotional responsibility when participating in deactivation decisions, particularly after long therapeutic relationships with patients and families. Shared protocols, multidisciplinary discussion, and clear institutional guidance may reduce this burden while reinforcing that deactivation represents an active clinical decision grounded in proportionality rather than therapeutic failure.

### 6.4. Ventricular Assist Devices and the Limits of Life-Sustaining Technology

Ventricular assist devices present additional ethical complexity because they may sustain life over prolonged periods while simultaneously exposing patients to infection, bleeding, repeated hospitalisations, dependence on external power sources, and progressive frailty [25,48,49].

For many patients these devices provide substantial survival and quality-of-life benefits. However, when recovery, transplantation, or long-term therapeutic goals are no longer achievable, continued support may prompt difficult questions regarding proportionality and the appropriate limits of life-sustaining technology.

Regular reassessment of treatment goals, transparent communication with patients and families, and early discussion of potential future deactivation are essential components of ethically robust device management. Ultimately, operational ethics seeks to ensure that cardiovascular technologies continue to serve the patient’s goals throughout the course of illness rather than allowing technological possibilities alone to determine the trajectory of care.

Although these therapies are frequently discussed together, ICD shock deactivation, withdrawal of pacing in pacemaker-dependent patients, CRT continuation, temporary mechanical circulatory support withdrawal, and durable LVAD deactivation represent ethically distinct clinical situations with different physiological consequences, legal considerations, emotional implications, and communication needs. For this reason, Operational Ethics emphasises anticipatory planning and device-specific ethical assessment rather than a uniform approach to all cardiovascular technologies. These distinctions are summarised in the revised Table 2.

## 7. Inequity and Hidden Moral Triage in the CICU

In this review, we use the term “hidden moral triage” to describe the influence of implicit bias, structural inequities, resource constraints, and undocumented or insufficiently explicit selection processes on decisions regarding access to advanced cardiovascular therapies. The term does not imply that all differences in treatment allocation reflect discrimination; rather, it highlights situations in which value-laden or contextual factors may influence clinical selection without being formally acknowledged within explicit triage criteria.

Decisions regarding CICU admission, therapeutic escalation, device implantation, and treatment limitation are primarily based on clinical severity, reversibility, expected benefit, comorbidity burden, neurological prognosis, technical feasibility, and resource availability. Nevertheless, observational evidence suggests that implicit structural factors—including age, sex, socioeconomic circumstances, communication barriers, and family advocacy—may also influence these processes. In this review, the term ‘hidden moral triage’ refers to the potential contribution of these less explicit factors to allocation decisions rather than implying that observed differences necessarily represent unconscious discrimination or ethically inappropriate care.

In contemporary cardiovascular critical care, allocation decisions increasingly rely on structured multidisciplinary assessment. Shock teams, standardised referral pathways, repeated reassessment, and explicit candidacy criteria aim to improve consistency when considering admission to the CICU or advanced mechanical circulatory support. Prognostic scores and risk models may contribute objective information regarding mortality risk, organ dysfunction, frailty, and neurological prognosis; however, they should support rather than replace individualised clinical judgement. Consequently, differences in treatment allocation frequently reflect genuine prognostic uncertainty, technical feasibility, institutional expertise, or resource availability, in addition to the potential influence of structural inequities. Operational Ethics should not only facilitate recognition of hidden sources of inequity but also promote practical mitigation strategies. These include the use of predefined eligibility criteria, multidisciplinary review of complex cases, structured documentation of treatment escalation or limitation decisions, standardised referral pathways, periodic audit of access to advanced therapies, and early involvement of palliative care and social work when appropriate. Examples of these strategies are summarised in Table 3.

### 7.1. Ageism: The Silent Gatekeeper

Age frequently operates as a covert criterion in decisions about advanced support. While frailty and biological age provide meaningful prognostic information, chronological age alone continues to influence clinical judgement. Patients above certain age thresholds may be implicitly excluded from ECMO, Impella^®^, or VAD, even when their functional status or personal values suggest potential benefit [50,51].

This constitutes a form of structural ageism, where assumptions about quality of life, recovery potential, or “appropriateness” override individualised assessment. Ethically, denying escalation based solely on age risks reducing the older patient to a demographic stereotype rather than a person with goals, history, and agency.

### 7.2. Gender Bias: Differences in Diagnosis, Escalation, and Moral Visibility

Gender inequities permeate acute cardiovascular care. Women are less likely to be admitted to high-intensity units, less likely to receive mechanical circulatory support, and more likely to have symptoms dismissed or interpreted through psychosocial lenses [52].

In the CICU, this manifests as differential thresholds for escalation: women may be perceived as “less severe” or “less suitable” candidates for ECMO or invasive shock therapies. Moreover, societal expectations of passivity or emotional expression may influence clinicians’ perceptions of resilience or “treatment worthiness.” [53,54,55,56].

### 7.3. Mental Health, Addiction, and the Stigma of “Low Adherence”

Patients with psychiatric conditions, cognitive or physical impairment, substance use disorders, or social instability often face moralised judgments that shape clinical decision-making. Labels such as “poor adherence,” “non-compliant,” or “difficult historian” may act as moral shortcuts, diminishing perceived deservingness of advanced support.

### 7.4. The Power of Family Advocacy and Social Capital

In the CICU, social visibility matters. Patients accompanied by assertive families, strong advocacy networks, or high cultural capital often receive more attention, more reconsideration, and more escalation. In contrast, patients who are socially isolated, or unable to express themselves clearly may receive less intensive care, not because of clinical futility but due to a deficit of social voice [57,58].

### 7.5. Socioeconomic and Geographic Disparities

Access to a tertiary cardiovascular centre, and therefore to a CICU bed, is deeply influenced by socioeconomic status (SES) and geography. Patients from underserved regions or without rapid transfer pathways may never be considered for ECMO or advanced shock therapies [59].

Once inside the system, lower SES may still affect expectations of adherence, perceived social worth, and the intensity of decision-making efforts [60,61,62].

### 7.6. The First Ethical Decision: Who Gets In

Perhaps the most ethically charged moment in cardiovascular critical care occurs before any device is activated: the allocation of the CICU bed itself. This threshold decision—who is accepted, who is transferred, and who is declined—determines who becomes eligible for the full ethical repertoire of modern medicine [63].

Because admission to the CICU determines access to advanced monitoring and life-sustaining therapies, these decisions have both clinical and ethical implications. Transparent documentation of the reasons supporting acceptance, transfer, non-escalation, or exclusion—including severity of illness, reversibility, expected benefit, technical feasibility, patient preferences, and resource availability—may improve consistency and facilitate ethical accountability. Such an approach does not replace clinical judgement but makes the reasoning underlying complex allocation decisions more explicit and open to multidisciplinary review.

## 8. Family Presence, Emotional Dynamics and Communication Under Pressure

The CICU is one of the few clinical environments where advanced technology, rapidly evolving physiology, and profound emotional uncertainty converge within minutes. Families frequently encounter life-threatening situations without prior knowledge of the therapies being offered, yet they are often invited to participate in decisions with immediate and potentially irreversible consequences [57,58]. In this context, communication becomes not only an ethical obligation but also a core clinical intervention that shapes trust, shared decision-making, and the quality of care [64].

### 8.1. Family Presence During Resuscitation and High-Risk Procedures

Whether family members should be present during cardiopulmonary resuscitation (CPR), reintubation, or other invasive procedures remains an area of ongoing ethical discussion. Supporters argue that family presence may promote transparency, facilitate grieving, and reinforce trust in the healthcare team, whereas concerns persist regarding emotional distress, misunderstanding of medical procedures, and the potential impact on healthcare professionals [40].

In the CICU, where resuscitation frequently occurs within the broader context of prolonged critical illness and advanced cardiovascular technologies, decisions regarding family presence should be individualised rather than protocol-driven. Careful preparation, appropriate staff support, and clear communication before, during, and after the event are essential to maximise potential benefits while minimising avoidable harm [40].

### 8.2. Emotional Escalation in a Technological Environment

The technological environment of the CICU inevitably shapes how families interpret the patient’s condition. Continuous monitoring, visible life-support devices, and repeated therapeutic interventions may reinforce the perception that ongoing technological activity necessarily implies continued therapeutic possibility.

Clinicians therefore face the complex task of balancing hope with realism, providing honest prognostic information while maintaining compassion and emotional support [64,65].

### 8.3. Communication Under Pressure: Consent When Time Disappears

Obtaining informed consent in the CICU frequently occurs under conditions of considerable time pressure, prognostic uncertainty, and emotional distress. Decisions regarding ECMO, invasive ventilation, treatment limitation, or withdrawal of life-sustaining therapies often cannot await prolonged deliberation.

Rather than viewing informed consent as a single event, contemporary critical care increasingly recognises consent as an ongoing process that evolves throughout the patient’s clinical trajectory. Initial decisions made during acute deterioration should therefore be revisited whenever clinical stability allows, ensuring that patients and families progressively develop a clearer understanding of prognosis, available treatment options, and goals of care [66,67] (Table 4).

### 8.4. The Double Vulnerability of Families and Clinicians

Communication in the CICU exposes not only the vulnerability of patients and families but also that of healthcare professionals. Cardiologists, intensivists, nurses, and trainees frequently navigate conversations involving uncertainty, potential treatment failure, and end-of-life decisions while simultaneously managing rapidly evolving clinical situations [68].

These circumstances may contribute to moral distress, particularly when clinicians perceive tension between what is technically feasible and what they believe represents the most proportionate course of care. Families may also experience uncertainty when confronted with changing prognostic information or differing interpretations of treatment goals. Structured communication strategies—including multidisciplinary family meetings, ethical huddles, pre-briefings, and post-event debriefings—may reduce these tensions by creating shared understanding among all participants.

### 8.5. Building Structured Communication Pathways

High-quality communication in the CICU should not depend solely on individual experience or communication skills. Instead, it should be supported by institutional structures that facilitate consistent, patient-centred decision-making [64]. Important elements include:

- Early identification of the patient’s surrogate decision-maker;
- Regular, structured family meetings at predictable intervals;
- Transparent discussion of prognosis, uncertainty, and treatment goals;
- Early integration of palliative care when appropriate;
- Documentation that captures not only clinical decisions but also the ethical reasoning supporting them.

Established communication frameworks such as SPIKES, REMAP, and the Serious Illness Conversation Guide may facilitate these conversations by providing reproducible approaches for discussing prognosis, uncertainty, patient values, and treatment goals [69].

## 9. Research, Consent and Vulnerability in the CICU

Research in the CICU is conducted in a clinical environment characterised by severe physiological instability, prognostic uncertainty, and frequent impairment of decision-making capacity. These same features that make cardiovascular critical care an essential setting for clinical research also create important ethical challenges regarding informed consent and participant protection.

Many patients are sedated, mechanically ventilated, delirious, or otherwise unable to provide fully informed consent at the time when research participation is considered. Consequently, investigators frequently rely on surrogate decision-makers or deferred consent procedures, both of which introduce additional ethical complexity. Family members are often asked to make research decisions while simultaneously coping with emotional distress, uncertainty, and rapidly evolving clinical situations.

The distinction between clinical care and research may also become less apparent in highly technological environments such as the CICU. When innovative therapies, advanced monitoring, or investigational interventions are delivered alongside routine clinical care, patients and families may overestimate the likelihood of direct personal benefit—a phenomenon commonly described as therapeutic misconception. Practical safeguards include deferred or staged consent when legally permitted, renewed consent after recovery of decision-making capacity, and clear separation between clinical recommendations and research invitations. These measures acknowledge that participant protection must continue throughout, rather than end with, initial enrolment.

From an operational perspective, research ethics should be integrated into routine CICU workflows rather than considered only at the time of study approval. Practical measures include systematic assessment of decision-making capacity, documentation of the consent process or justification for deferred consent when permitted, periodic reassessment of ongoing participation as the patient’s clinical condition evolves, multidisciplinary discussion of ethically complex enrolment decisions, and transparent communication with patients and families whenever feasible. Embedding these practices into everyday clinical routines reinforces ethical accountability while facilitating responsible research in critically ill cardiovascular populations.

## 10. Moral Distress in the CICU Team and the Need for Ethical Structures

The CICU is not only a site of physiological complexity but also an environment in which clinicians frequently encounter ethically demanding situations. Moral distress may arise when healthcare professionals perceive tension between what they believe represents the most appropriate course of action and what is ultimately delivered because of clinical uncertainty, organisational constraints, technological momentum, or differing expectations among stakeholders.

These situations may occur when advanced therapies appear to prolong treatment without a realistic prospect of recovery, when uncertainty regarding prognosis persists, or when disagreement exists between members of the healthcare team or with patients’ families. Hierarchical structures may further complicate these situations by making junior physicians, nurses, or trainees less likely to express ethical concerns openly. Repeated exposure to emotionally demanding situations may contribute to compassion fatigue, burnout, and reduced professional well-being [70].

Moral distress should therefore be approached as an organisational concern rather than solely as an individual resilience problem. Practical institutional responses are summarised in Table 5. Similar experiences have been described among critical care nurses, perfusionists, and ECMO specialists, reinforcing that moral distress represents a multidisciplinary phenomenon rather than a physician-only concern. From an operational perspective, addressing moral distress requires institutional processes rather than relying solely on individual resilience. Practical interventions include routine ethics debriefings following ethically challenging cases, standardised documentation templates for treatment escalation or limitation decisions, timely access to clinical ethics consultation, structured multidisciplinary support after withdrawal of mechanical circulatory support, and formal mechanisms enabling nurses, trainees, and other team members to raise ethical concerns without fear of hierarchy or reprisal. These measures promote organisational learning, improve transparency, and help integrate ethical reflection into routine CICU practice.

## 11. Toward an Operational Ethics for the CICU

The themes explored throughout this review highlight a central challenge of contemporary cardiovascular critical care: technological capabilities have advanced rapidly, whereas the practical integration of ethical reasoning into routine bedside decision-making remains less developed.

Operational ethics does not replace existing ethical frameworks or professional guidelines. Rather, it provides a practical approach for embedding ethical reflection within the everyday workflows of the CICU, allowing ethical reasoning to accompany clinical action rather than follow it.

In practice, the framework can be incorporated into existing CICU processes at predefined decision points: before major escalation, during daily multidisciplinary review, at the end of a time-limited trial, when goals of care change, and after ethically challenging events. Table 5 summarises the proposed triggers, operational tools, and bedside questions.

Although many examples discussed in this review are based on European guidelines and cardiovascular critical care practice, the principles underlying Operational Ethics are not intended to be jurisdiction-specific. The proposed framework is sufficiently flexible to support bedside ethical deliberation across healthcare systems with different legal frameworks, organisational structures, resource availability, and cultural approaches to end-of-life decision-making. The specific implementation of treatment limitation, withdrawal of life-sustaining therapies, organ donation, and allocation of advanced cardiovascular support should always remain consistent with local legislation, institutional policies, and societal values.

These tools are intended to complement—not replace—clinical judgement, shared decision-making, palliative care, formal ethics consultation, or existing professional guidance. Their feasibility, acceptability, and effect on clinical and patient-centred outcomes require prospective evaluation.

Future evaluation of Operational Ethics should proceed prospectively and in stages. Initial studies should assess feasibility, acceptability, adherence to predefined ethical checkpoints, completeness of documentation, and interprofessional reliability in identifying ethically relevant triggers. Subsequent studies could evaluate patient- and family-reported communication outcomes, use and timing of ethics or palliative care consultation, clinician moral distress, consistency of treatment-escalation decisions, and potential differences in access to advanced therapies. These outcomes would allow the feasibility, reliability, construct validity, and potential clinical impact of the framework to be examined before considering broader implementation.

## 12. Conclusions

The contemporary CICU combines advanced technologies, prognostic uncertainty, and recurrent decisions regarding escalation, treatment limitation, device management, communication, and allocation of specialised resources. These decisions require ethical reasoning that can operate within, rather than separately from, routine clinical care.

Operational Ethics is proposed as an implementation-oriented framework that organises established ethical principles into structured pause points, multidisciplinary reassessment, communication, and documentation. It should be regarded as a conceptual model whose feasibility, acceptability, implementation, and clinical impact should be prospectively evaluated across different healthcare settings.

## Figures and Tables

**Table 1:** Main ethical domains in the CICU.

Domain	Description	Typical Scenarios
High-intensity technology	Rapid access to advanced support that can be escalated quickly	ECMO activation, emergent reintubation for clinical decline
Time-compressed decisions	Decisions often must be made within minutes, limiting reflection	Shock, cardiac arrest, peri-resuscitation interventions
Prognostic uncertainty	Difficulty predicting neurological, multiorgan or cardiac recovery	Post-arrest syndrome, shock of unclear aetiology
Futility and proportionality	Risk of continuing non-beneficial, burdensome interventions	Prolonged ECMO, recurrent Impella^®^ escalation, persistent VAD complications
Device-related ethics	Moral complexity of initiating, withholding or withdrawing devices	ICD deactivation, generator replacement, VAD end-of-life
Structural inequity	Biases in access, triage and continuation of care	Age, gender, SES, mental health, advocacy power
Communication under pressure	Difficult discussions in unstable or rapidly changing situations	DNR, non-escalation, withdrawal of support
Family dynamics & conflict	Divergent expectations between families and clinicians	Requests for maximal therapy, disagreement over goals-of-care
Moral distress	Staff experiencing burden from perceived disproportionate care	Prolongation of dying, family-driven escalation
End-of-life transitions	Ethical complexity at discontinuation of support	Controlled donation after circulatory death (DCD)

**Table 2:** Ethical dilemmas associated with advanced mechanical circulatory support and implantable devices in acute cardiovascular care.

Device/Therapy	Main Ethical Issue	Preferred Timing of Discussion	Operational Ethics Considerations
ICD shock therapy	Shock deactivation	Advance care planning; disease progression	Clarify goals of care and document shared decision-making before deactivation.
Pacemaker-dependent patients	Withdrawal in pacing dependence	Disease progression or end-of-life discussions	Evaluate proportionality, patient preferences, and anticipated physiological consequences.
CRT	Generator replacement or continuation	Elective replacement	Reassess whether ongoing therapy remains aligned with prognosis and patient goals.
Temporary MCS (ECMO, Impella^®^, IABP)	Withdrawal after unsuccessful support	Before implantation and during predefined reassessment	Establish therapeutic goals before initiation and predefined criteria for continuation or withdrawal.
Durable LVAD	Elective deactivation	Progressive multisystem failure or patient request	Multidisciplinary discussion, advance care planning, family communication, and documentation of ethical reasoning.

**Table 3:** Structural inequities and hidden triage factors influencing decisions in the CICU.

Potential Source of Inequity	Potential Consequence	Operational Ethics Mitigation Strategy
Advanced age	Reduced access to advanced therapies despite potential benefit	Use predefined eligibility criteria, document reasons for non-escalation, multidisciplinary review.
Sex and gender bias	Under-recognition or delayed referral	Promote standardised diagnostic pathways and periodic audit of referral patterns.
Socioeconomic vulnerability	Reduced access to specialised care	Early involvement of social work, transparent referral pathways, documentation of barriers.
Family advocacy/social capital	Unequal representation of patient preferences	Use structured family meetings and standardised documentation regardless of family dynamics.
Resource constraints	Variable allocation decisions	Apply explicit allocation criteria, multidisciplinary discussion, and regular review of institutional practices.

**Table 4:** Communication challenges in high-stakes encounters in the CICU.

Clinical Situation	Ethical Objective	Operational Approach	Suggested Documentation/Communication
Initiation of advanced MCS	Define proportional therapeutic goals	Establish goals before implantation	“Treatment initiated as a bridge-to-decision with predefined reassessment after 72 h.”
Daily reassessment	Reassess proportionality	Multidisciplinary review	“Goals of care reviewed; current treatment remains consistent with patient’s prognosis and preferences.”
Time-limited trial	Evaluate response	Structured reassessment	“Predetermined clinical objectives achieved/not achieved; continuation of support discussed by the multidisciplinary team.”
Treatment limitation	Ensure transparency	Shared decision-making	“Decision based on proportionality, patient values, multidisciplinary consensus, and documented discussion with family.”
Withdrawal of life-sustaining treatment	Ethical continuity of care	Palliative transition	“Withdrawal discussed with patient/surrogate; comfort-focused care initiated in accordance with agreed goals of care.”

**Table 5:** Operational ethics toolkit for routine CICU practice.

Clinical Situation	Operational Ethics Tool	Key Bedside Question(s)	Suggested Action
Initiation of advanced mechanical circulatory support (ECMO, Impella^®^, IABP)	Ethical Pause Point	What is the therapeutic goal (recovery, bridge to decision, bridge to transplant/LVAD, or palliation)? Is the expected benefit proportionate to the anticipated burden?	Define the treatment objective before initiation and document criteria for reassessment or discontinuation.
Daily multidisciplinary rounds	Structured Ethical Checkpoint	Does the current treatment remain aligned with the patient’s prognosis, goals of care and previously agreed therapeutic plan?	Reassess proportionality, update goals of care and document any changes in the clinical record.
Time-limited trial of advanced support	Predefined Reassessment	Have the predefined clinical objectives been achieved? Is continuation still justified?	Continue, modify or withdraw support according to previously established criteria and multidisciplinary discussion.
ICD, pacemaker or VAD replacement/deactivation	Goals-of-Care Review	Does continued device therapy remain consistent with the patient’s prognosis, preferences and expected quality of life?	Discuss replacement or deactivation proactively and document shared decision-making before elective procedures whenever possible.
AI-supported prediction or candidacy assessment	Algorithmic Accountability Check	Is the model validated for this clinical context? Is its recommendation interpretable, applicable to this patient and consistent with bedside clinical judgement?	Use AI as decision support only; maintain clinician responsibility for the final decision and document disagreements between algorithmic output and clinical assessment when relevant.
Family meetings and major treatment decisions	Structured Communication Pathway	Has prognosis, uncertainty and the rationale for treatment decisions been clearly explained and understood?	Identify surrogate decision-makers, provide regular updates, revisit discussions as the clinical situation evolves and document key conversations.
Treatment limitation or withdrawal	Multidisciplinary Micro-deliberation	Does continued treatment remain proportionate? Are patient values, previously expressed preferences and family perspectives adequately considered?	Confirm consensus whenever possible, document the ethical rationale and ensure continuity of symptom-directed care.
After ethically challenging cases	Ethics Debriefing	What ethical challenges emerged? Could future decision-making be improved?	Conduct brief multidisciplinary debriefings, identify system improvements and promote organisational learning.
Research enrolment in critically ill patients	Research ethics checkpoint	Does the patient have decision-making capacity? If not, is deferred consent ethically and legally appropriate?	Document consent process, reassess participation, involve surrogates when appropriate, and review complex cases within the multidisciplinary team.
Ethically challenging case or withdrawal of advanced support	Ethics Debriefing	Has the team identified unresolved ethical concerns or sources of moral distress?	Conduct a structured multidisciplinary debriefing, document lessons learned, facilitate access to ethics consultation when appropriate, and encourage all team members—including nurses and trainees—to raise concerns.

Abbreviations: AI, artificial intelligence; CICU, Cardiovascular Intensive Care Unit; ECMO, extracorporeal membrane oxygenation; IABP, intra-aortic balloon pump; LVAD, left ventricular assist device; VAD, ventricular assist device.

## Data Availability

No new data were created or analysed in this study. Data sharing is not applicable to this article.

## Abbreviations

CICU	Cardiovascular Intensive Care Unit
CPR	Cardiopulmonary Resuscitation
CRT	Cardiac Resynchronization Therapy
DCD	Donation after Circulatory Death
DNR	Do Not Resuscitate
ECMO	Extracorporeal Membrane Oxygenation
ERC	European Resuscitation Council
IABP	Intra-Aortic Balloon Pump
ICD	Implantable Cardioverter–Defibrillator
ICU	Intensive Care Unit
MCS	Mechanical Circulatory Support
SES	Socioeconomic Status
VA-ECMO	Veno-Arterial Extracorporeal Membrane Oxygenation
VAD	Ventricular Assist Device
VV-ECMO	Veno-Venous Extracorporeal Membrane Oxygenation
